# The oral microbiome: A Lesson in coexistence

**DOI:** 10.1371/journal.ppat.1006719

**Published:** 2018-01-25

**Authors:** Ahmed S. Sultan, Eric F. Kong, Alexandra M. Rizk, Mary Ann Jabra-Rizk

**Affiliations:** 1 Department of Oncology and Diagnostic Sciences, Dental School, University of Maryland, Baltimore, Maryland, United States of America; 2 Department of Microbiology and Immunology, School of Medicine, University of Maryland, Baltimore, Maryland, United States of America; 3 Graduate Program in Life Sciences, Molecular Microbiology and Immunology Program, University of Maryland, Baltimore, Maryland, United States of America; McGill University, CANADA

## Introduction

Whether in human, animal, or microbial communities, the resolve to coexist is the quintessence of survival. Therefore, in natural habitats where resources are limited, individual species must collaborate with others in order to survive and endure [1]. Yet even among the highest of species, harmonious coexistence is often elusive, plagued by power struggles, competition, and opportunism. The human mouth with its various niches is an exceptionally complex habitat, harboring unique and diverse microbial communities [2, 3]. In addition to being the initiation point of digestion, the oral microbiome is crucial in maintaining oral health [3–5]. The ecological balance in the oral cavity is maintained through antagonistic as well as mutualistic interspecies interactions [3]. However, perturbations that disrupt the equilibrium of this ecosystem may lead to the overgrowth of species with pathogenic potential and, in turn, the development of oral disease [6]. Although recent advances in molecular biology have facilitated analyses of the oral microbiome, there is a great deal we do not understand about its functions and the processes underlying the transition from a healthy oral microbiome to a disease-associated microbiome. Here, we highlight some of the host and microbial factors orchestrating the ecological balance in the oral cavity, crucial for maintaining a healthy oral microbiome.

## The bacterial microbiome: A known known

**Fig 1:**
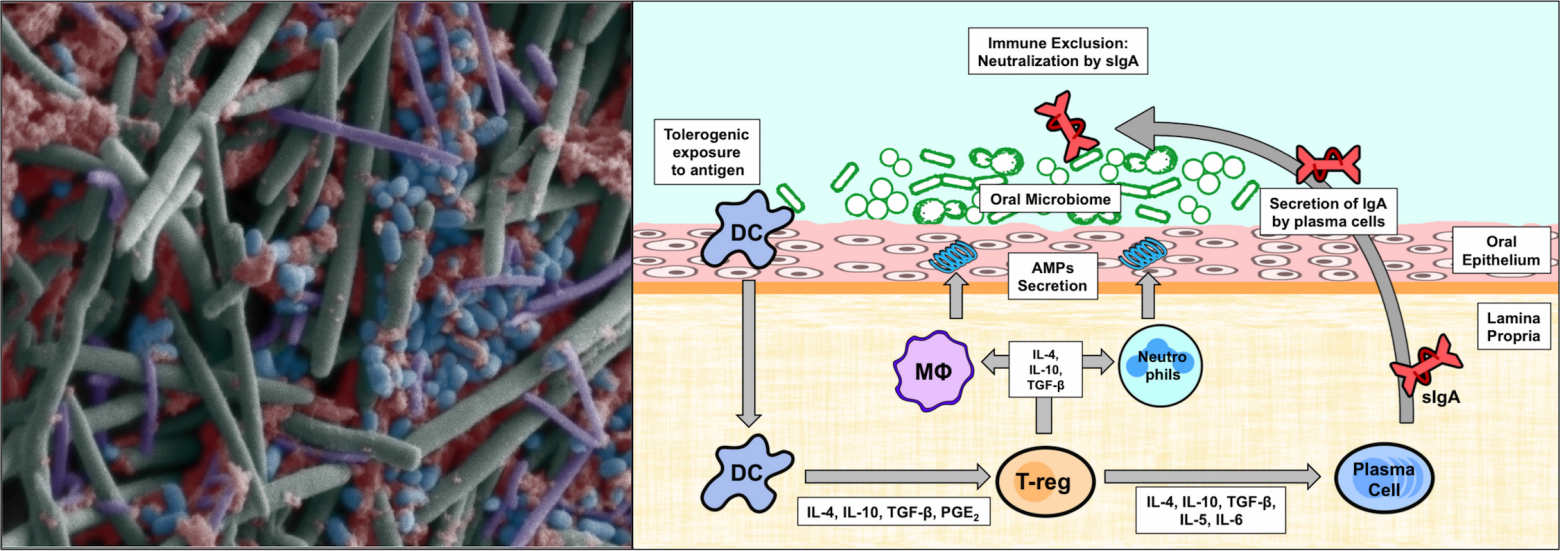
Complexity of the oral microbial flora and host immune responses. (A) A false-colored scanning electron image demonstrating the diversity of the microbial population residing in the deep subgingival pockets where the environment is anaerobic. (B) A schematic illustrating the complexity of the host immune responses involved in maintaining homeostasis in the oral cavity. Under tolerogenic conditions, resident DCs sample the microbial species in the millieu. In the absence of damage and infection, this recognition of microbial antigens or PAMPs results in the secretion of immunosuppressive cytokines and growth factors by the DC. This cytokine profile induces the differentiation of T-regs, which release a series of cytokines to further maintain the tolerogenic state. B cells, which mature into plasma cells, produce sIga, which neutralizes microorganisms in a noninflammatory manner to limit colonization, a process termed “immune exclusion.” Additionally, T-regs also induce a tolerogenic state in resident MΦs and neutrophils, triggering the release of AMPs into the microenvironment. AMP, antimicrobial peptide; DC, dendritic cell; IL, interleukin; MΦ, macrophage; PAMP, pathogen-associated molecular pattern; PGE_2_, Prostaglandin E2; sIga, secretory immunoglobulin A; T-regs, T regulatory cells; TGF-β, transforming growth factor beta.

The healthy human mouth is one of the most heavily colonized parts of our bodies, containing hundreds of different bacterial, viral, and fungal species [3]. There is a conserved oral microbial community in healthy mouths at the genus level; however, despite commonalities, microbial diversity is individual-specific and site-specific [3]. Although the buccal and palatal mucosae are areas with low microbial diversity, the tongue is highly papillated with some anaerobic sites and therefore harbors more diverse microflora, including anaerobes [3, 5]. In contrast, the teeth enable large masses of microbes to accumulate as biofilms known as plaque. Obligate anaerobes, such as *Porphyromonas*, *Fusobacterium*, *Prevotella*, and *Treponema*, primarily reside in gingival crevices or periodontal pockets where the environment is anaerobic (Fig 1A) [3, 5]. However, oral surfaces are subjected to constant environmental changes, which may lead to changes in the microflora [3]. Indigenous oral bacteria produce a range of extracellular factors such as adhesins that promote social networking; for example, *F*. *nucleatum* expresses adhesins that recognize streptococci and a lectin that interacts with *Porphyromonas gingivalis* [5]. Furthermore, communication or quorum sensing between bacteria via small, secreted signaling molecules is fundamental to social evolution [3].

The interactions within mixed-species microbial communities can be synergistic in that the presence of one microorganism generates a niche for another, and a product secreted by one species serves as nutrient for another; as an example, lactic acid produced by streptococci is used as a carbon source by veillonellae [3]. Although cross feeding among the microbial residents can be mutually beneficial, microbial metabolic activity can modify the oral environment and induce microbial selection to create a more pathogenic microbiome. Therefore, the microbial consortia are locked in metabolic communication, and it is therefore imperative to take a metabolomic approach in analyzing the oral microbiome [7].

## The mycobiome: A known unknown

While the oral bacteriome is increasingly well characterized, the fungal microbiome or mycobiome is a new and poorly recognized biome. Research on oral fungi has traditionally centered on the opportunistic fungal species *Candida albicans*, which, although a normal inhabitant of the oral mucosa, can rapidly transition to a pathogen [8]. Recent DNA-based studies of the oral mycobiome, however, have revealed a vast number of fungal species as potential oral residents [9, 10]. Taking a pyrosequencing approach, a landmark study profiling the “basal” healthy oral mycobiome identified 85 genera and over 100 different commensal fungal species [10]. Although *Candida* species were the most prevalent, three other genera—*Aspergillus*, *Fusarium*, and *Cryptococcus*, known to be pathogenic in humans—were also frequently isolated, warranting further investigations to unravel the role fungi play in oral homeostasis and disease [9–11]. One caveat in studying the mycobiome, however, is the possibility that some identified non-*Candida* fungi may be contaminants rather than integrated members of the mycobiome.

Interestingly, as cocolonizers in the oral cavity, streptococci have been shown to provide *C*. *albicans* with a carbon source for growth, as well as provide adhesion sites for *C*. *albicans* to persist within the oral cavity [5]. Furthermore, by utilizing the lactic acid created by streptococci as a carbon source, *C*. *albicans* lowers oxygen tension levels, which is advantageous to *Streptococcus gordonii* [12]. This mutualistic fungal–bacterial relationship may, however, have repercussions to the host; using a rat model, studies have indicated that the avid co-adherence between *C*. *albicans* and the cariogenic bacterial species *S*. *mutans* may enhance the development of dental caries [13]. Significantly, recent in vitro studies demonstrated that, during mixed biofilm growth, *C*. *albicans* conferred the bacterial pathogen *Staphylococcus aureus* with protection against antimicrobials [14]. Although the mechanism was shown to involve fungal cell wall–secreted polysaccharides in the biofilm matrix, quorum sensing or cell–cell communication mediated by secreted molecules was also shown to contribute to the enhanced bacterial drug tolerance [15]. Whether similar processes occur within fungal–bacterial biofilms in the oral cavity, however, remains to be investigated. Combined, these studies clearly signify that fungi represent a significant component of the oral microbiome and therefore should not be excluded from future oral microbiome studies.

## Keeping the peace

As the oral mucosa provides a favorable niche for many microbial species, it becomes the responsibility of the host immunity to differentiate pathogen from commensal. Colonization itself is a harmless state; however, the fact that, under certain conditions, some microbial residents can turn pathogenic presents a quandary for oral immunity because the question is no longer how to differentiate friend from foe but rather how to determine when a friend becomes a foe. Therefore, setting the “rules of engagement” by the host immune system is an exceedingly complex and dynamic task. In essence, the immune responses in the oral cavity need to be naturally oriented towards a more tolerogenic state, which has led to the assertion that the oral mucosa is an immune-privileged site [16]. Mounting an aggressive immune response against colonizing microbes that pose no threat would be unnecessary, metabolically wasteful, and potentially damaging to host tissues.

The oral mucosa maintains resident dendritic cells, which act as antigen-presenting cells (APCs) releasing proinflammatory cytokines that activate adaptive immunity (Fig 1B) [16]. However, the mucosal dendritic cells are predisposed to a tolerogenic state, resulting in the secretion of anti-inflammatory immunomodulators such as interleukin 10 (IL-10), transforming growth factor beta (TGF-β), and Prostaglandin E2 [17]. These effectors act in suppressing the activity of the immune system and generating T regulatory cells (T-regs) in the tissue, thereby propagating a tolerant state [18]. Although it is not well understood how mucosal dendritic cells induce a state of tolerance, some studies have cited the role of immune exhaustion, in which APCs are no longer activated in response to specific commensal antigens, essentially becoming desensitized [17]. While the immune interactions of pathogens with the oral mucosa remain ambiguous, the pathogen-associated molecular patterns (PAMPs) of normal mucosa do not trigger an inflammatory response. Unfortunately, the expression and character of these PAMPs do not necessarily change during a pathogenic shift because the transition is not always associated with novel antigenic features in some pathogens. However, the expression of virulence factors such as adhesins and enzymes does remain a hallmark of pathogenesis and aids in the detection of commensal-turned-pathogen for the immune system. Overall, the detection of commensal from pathogen at mucosal tissues remains a highly complex and nuanced dynamic.

Other secondary and local oral immune effectors play an important supportive role in providing mucosal protection, such as the salivary antibody secretory immunoglobulin A (sIgA), which acts in noninflammatory mediated neutralization of microbes, a phenomenon termed “immune exclusion” [19]. Additionally, antimicrobial peptides (AMPs), most notably histatins, defensins, and cathelicidin LL-37, often constitute the first line of defense against microbes in the oral cavity and can interact synergistically in limiting microbial colonization [20]. In essence, maintenance of oral health reflects the continuous negotiations between resident inflammatory immune cells and the microbial ecology in the oral cavity.

## Microbial insurgency

While oral microorganisms exist in a symbiotic capacity, maintaining relationships with the host based on mutual benefits, some can transition to pathogens when they breach the barrier of commensalism, causing disruption of oral homeostasis, or “dysbiosis” [3, 5]. Despite advances in our knowledge of the healthy oral microbiome, the functional aspects that lead to dysbiosis remain largely unknown [6]. What is now clear, however, is that oral diseases arise as a result of a change in the proportion of certain species with greater pathogenic potential within the indigenous flora [5]. This change in the “commensal” microbiota is accompanied by disruption of the host immune homeostasis and development of an inflammatory response. Therefore, it is the prevalence of a certain combination of microbial species coupled with the inability of the host to contain their proliferation that is more indicative of a risk to develop disease. Consequently, the study of microbial pathogenesis in oral disease has shifted focus from the study of single bacterial species to the study of the ecology and virulence of polymicrobial communities [5].

Of the 40% of the bacterial species that have been cultivated, approximately 10 have been recognized to have pathogenic potential, most of which are gram-negative anaerobic bacteria that flourish in subgingival pockets such as *P*. *gingivalis*, *T*. *denticola*, *F*. *nucleatum*, and *Prevotella* sp. (Fig 1A) [3, 21]. Accumulation of these microbial populations within the dysbiotic community induces inflammation, causing destruction of oral tissue. This process is best exemplified by periodontitis, which is hypothesized to be a “microbial-shift disease” in which the microbial populations shift from gram-positive aerobic bacteria to gram-negative anaerobes [2, 3, 22]. Interestingly, even at low levels, *P*. *gingivalis* can cause a change in the quantity and composition of the microbial flora, resulting in an inappropriate inflammatory reaction to the normal microbiota [2]. Moreover, studies have indicated that *C*. *albicans* can exacerbate periodontitis through enhancing *P*. *gingivalis* invasion, and an increase in *C*. *albicans* colonization has been associated with severity of periodontitis [23, 24].

Of more significance, the chronic oral inflammatory insult mediated by host immune responses has far-reaching implications for systemic health [25]. Recent evidence has indicated associative pathophysiological links between oral inflammatory diseases and systemic diseases such as diabetes, cardiovascular disease, and rheumatoid arthritis [26]. However, establishing a true causal link between oral inflammatory and systemic diseases is a tremendously onerous task, hindered by shared risk factors. What is now abundantly clear is that the trillions of bacteria constituting the microbiome are not passive bystanders and may be playing a critical role in systemic disease. However, more research is needed to develop strategies to target the dysbiotic mechanisms and improve oral health.

## Negotiating coexistence

Cooperation within shared societies is favored when there is a shared selfish interest in doing so. The oral microbiota has coevolved with us and has become an integral part of who we are [3]. In addition to metabolism and the establishment of mucosal immunity, one beneficial function of the microbiota is protection against foreign microbial species by occupying available niches, a concept known as “bacterial interference” or “colonization resistance” [27]. A recent study described a unique social organization by specific types of bacterial species within a microbial community with coordinated roles, initiating antagonistic actions to prevent the integration of a nonindigenous bacterial species [27]. Several mechanisms have been proposed to explain the colonization resistance, including stimulating the host immune response against invaders, competition for substrates and host binding sites, and generating a microenvironment that is inhibitory to potential competitors [27]. Therefore, colonization resistance has a significant role in the stability of established communities and is a crucial component of host defense against pathogens [27].

The notion that the microbiota can be manipulated to the host's advantage has been the focus of much research [27]. However, dissecting the biological properties that confer stability in the microbiome has been extremely difficult to elucidate due to the enormous microbial diversity. Uncovering the nuances of the concerted efforts of the microbiota and the host to live in relative harmony and sustain survival can perhaps be best revealed by the words of Lyndon B Johnson: "If we are to live together in peace, we must come to know each other better."
